# Response of Human Osteoblast to n-HA/PEEK—Quantitative Proteomic Study of Bio-effects of Nano-Hydroxyapatite Composite

**DOI:** 10.1038/srep22832

**Published:** 2016-03-09

**Authors:** Minzhi Zhao, Haiyun Li, Xiaochen Liu, Jie Wei, Jianguo Ji, Shu Yang, Zhiyuan Hu, Shicheng Wei

**Affiliations:** 1 CAS Key Laboratory for Biomedical Effects of Nanomaterials & Nanosafety, National Center for Nanoscience and Technology, Chinese Academy of Sciences, Beijing 100190, China; 2 Center for Craniofacial Stem Cell Research and Regeneration, Department of Orthodontics, Laboratory of Interdisciplinary Studies, Peking University School and Hospital of Stomatology, Beijing 100081, China; 3 State Key Laboratory of Protein and Plant Gene Research, College of Life Sciences, Peking University, Beijing 100871, China; 4 Center for Biomedical Materials and Tissue Engineering, Academy for Advanced Interdisciplinary Studies, Peking University, 100871, Beijing, China; 5 Key Laboratory for Ultrafine Materials of Ministry of Education, East China University of Science and Technology, Shanghai 200237, China

## Abstract

Nano-sized hydroxyapatite (n-HA) is considered as a bio-active material, which is often mixed into bone implant material, polyetheretherketone (PEEK). To reveal the global protein expression modulations of osteoblast in response to direct contact with the PEEK composite containing high level (40%) nano-sized hydroxyapatite (n-HA/PEEK) and explain its comprehensive bio-effects, quantitative proteomic analysis was conducted on human osteoblast-like cells MG-63 cultured on n-HA/PEEK in comparison with pure PEEK. Results from quantitative proteomic analysis showed that the most enriched categories in the up-regulated proteins were related to calcium ion processes and associated functions while the most enriched categories in the down-regulated proteins were related to RNA process. This enhanced our understanding to the molecular mechanism of the promotion of the cell adhesion and differentiation with the inhibition of the cell proliferation on n-HA/PEEK composite. It also exhibited that although the calcium ion level of incubate environment hadn’t increased, merely the calcium fixed on the surface of material had influence to intracellular calcium related processes, which was also reflect by the higher intracellular Ca^2+^ concentration of n-HA/PEEK. This study could lead to more comprehensive cognition to the versatile biocompatibility of composite materials. It further proves that proteomics is useful in new bio-effect discovery.

Polyetheretherketone (PEEK) is a semi-crystalline thermoplastic with excellent mechanical properties, high temperature durability, and good chemical and fatigue resistance. Being one of the most advanced engineering thermoplastics currently available, it has broad applications in the aerospace and marine industries. For biomedical applications, PEEK offers additional benefits including its ability to be repeatedly sterilized and shaped readily by machining and heat contouring. In recent years, PEEK polymer has made great inroads into biomedical field, particularly in the area of load-bearing orthopedic applications^1^ and dental applications^2^. It is regarded as bio-inert^3^ and hydrophobic in the bulk state and is often used as substrate material^4^. Modification of PEEK to obtain the bioactivity has been the interest of many researchers^5^,^6^,^7^,^8^. For bone implant materials, the aim is to accelerate osteoblast adhesion and proliferation, in order to promote osteointegration. Most of bioactive PEEK composites are prepared by compounding the PEEK with bioactive components. Hydroxyapatite (HA) is widely used in biomedicine as a bioactive material with the ratio of calcium to phosphorus similar to that of the natural bone^9^. Thus, HA/PEEK composition has been reported in a series of PEEK based composite materials^5^,^10^,^11^,^12^,^13^. These HA/PEEK composites have shown great promise as bioactive implants^4^,^13^. Melt mixing and injection molding have been a common method reported in the literature to produce HA/PEEK composites with HA fractions of up to 40% by weight^14^. Yu *et al*., reported that bioactivity of HA/PEEK composite increases with increasing HA volume fraction in the composite^10^. A histological study of animal model to PEEK composite was developed for HA/PEEK composite with high HA loading. Osteoblastic activities were seen in the formation of osteoid and osteocytes within lamellar bone in developing mature bone at longer implantation periods^14^.

Nano-sized biomaterial has drawn great attention in the biomedical field. It is reported that nano-sized hydroxyapatite has many bio-active functions better effect on promotion of cell attachment, cell growth and inhibition of cell apoptosis than micro-sized hydroxyapatite^15^,^16^. Implants made of PEEK nano-composites have a number of advantages such as increased bioactivity and better mechanical properties^17^,^18^. Nano-sized hydroxyapatite/PEEK composite could promote the functions of cells, including cell attachment, spreading, proliferation, alkaline phosphatase activity, calcium nodule formation, and expression of osteogenic differentiation-related genes^19^. The biological side effects and safety have to be taken into consideration for successful biomedical applications of any nano-composite materials. Nano-particles of certain sizes and surface area may cause unintended consequence/toxicity. A significant decrease in cell population was found after adding the HA nanoparticles to the osteoblasts^20^, as well as a time-dependent toxicological effects of HA nanoparticles on the pulmonary surfactants^21^. Even so, nanoparticles are not generally used alone in the implants and they usually play the role of addition agent in the composite materials. Therefore, bioactivity and toxicity both should be assessed for nanoparticles alone and final composite composition. PEEK has been reported to possess good biological interaction even without the addition of traditionally bioactive components^22^. Whether the composition of PEEK with nano-sized HA (n-HA) could reduce the adverse effects of free n-HA, and bring other desired performance need to be evaluated cautiously.

Bioactivity has many forms of expression. For bone implant materials, it can be the ability of inducing the precipitation of HA in simulation body fluid (SBF), differentiation potential of the osteoblast, promotion of new bone formation and so on. Toxicity, bioactivity and biocompatibility are all considered as biological effects, which need to be presented and evaluated in various levels and presentation. It is essential to understand the biological effects in-depth, and find the mechanisms of cell responses to material’s stimulation at global molecular level. In this study, HA/PEEK composite with 40% nano-sized HA was investigated to study the effect of high level HA on osteoblast’s cellular response at whole protein level. High throughput technologies have been explored to investigate biological effect of variety of biomaterials in the previous work of our group^23^,^24^. In this manuscript, a quantitative proteomic approach, so called stable isotope labeling with amino acids in cell culture (SILAC), was used to study the biological effect of HA/PEEK nano-composite (n-HA/PEEK) on human osteoblasts from the proteome aspect.

## Materials and Methods

### Materials and Characterizations

The nano-hydroxyapatite (HA) was synthesized in our laboratory via a hydrothermal treatment method. n-HA particles with a diameter of 23 ± 5 nm, a length of 47 ± 14 nm, and crystallinity of 85% ± 5% were obtained^23^ by a chemical precipitation method. Briefly, Ca(NO_3_)_2_ ⋅ 4H_2_O and (NH_4_)_2_HPO_4_ were dissolved in deionized water separately according to a Ca/P molar ratio of 1.67/1. The pH of each solution was adjusted to 9–10 with ammonia solution. Ca (NO_3_)_2_ solution was then dropped into the solution of (NH_4_)_2_HPO_4_ under continuous stirring. When this reaction was completed, hydroxyapatite was kept at room temperature for 24 hours, and the precipitate was obtained after washing with deionized water four times. Implantable grade PEEK (450 G, victrex, Invibio, Thornton Cleveleys, UK) and HA were mixed in a mixer at 60:40% weight ratio. The mix was then melt-extruded in a twin-screw extruder (Model TS1-30A, L:D = 30:1, Nanjing Reiya Co., China). A screw speed of 200 rpm and temperature ranges (180 °C to 245 °C) were used so that the composite raw materials could melt and mix sufficiently. The pellets were molded into rod-like samples (n-HA/PEEK) using an injection molding machine (Model: PS40E5ASE, NSISE, Japan). Using a wire cutting process, disc samples with 15 mm in diameter (the same with a well of 24-well plate) and 2 mm in thick were prepared for the cellular experiment in 24-well plates, and polished with a series of increasing SiC abrasive papers (80, 400 and 800 grit). They were ultrasonically cleaned in acetone, absolute ethanol and de-mineralized water, and then dried in open air. Pure PEEK disc samples were prepared via the same process.

The surface morphology of the samples was assessed by environmental scanning electron microscope (ESEM; Quanta 200FEG, Hillsboro, OR). X-ray diffractometer (Rigaku DMAX 2400) with Cu Kα radiation was used for identification of the phases. Fourier transform infrared spectroscopy (Magna-IR 750, Nicolet, ThermoFisher Scientific, Madison, WI) was used for detection of the bands and functional group of the materials in 4000-400 cm^−1^ wave number range. Surface wettability was evaluated by measuring the contact angle of distilled water on material surface using a CAS system (Data physics OCA20, Germany). The inductively coupled plasma atomic emission spectrometry (Leeman, Profile ICP-AES) was used to measure the concentration of calcium. The extraction medium were prepared using DMEM serum free medium as the extraction medium with the surface area of extraction medium ratio 3 cm^2^/mL in a humidified atmosphere with 5% CO_2_ at 37 °C for 14 days. PBS was set as reference medium. 6 piece of samples were used in the characterizations described above.

### Cell Culture, Cell Morphology, Adhesion, Proliferation and Alkaline Phosphatase (ALP) Activity

Human osteoblast-like MG-63 cells (CRL1427, ATCC, Manassas, VA) were seeded onto the PEEK and n-HA/PEEK samples in 24-well plates (made up of polystyrene). The culture medium was Dulbecco’s Modified Eagle Medium (DMEM) (Hyclone, Logan, UT) containing 10% (v/v) fetal bovine serum (FBS) (Gibco BRL, Grand Island, NY), penicillin (100 U/ml), and streptomycin sulfate (100 mg/ml). Cells were incubated at 37 °C with 5% CO_2_. For the evaluation of cell attachment, cells were cultured in 24-well culture plates at an initial seeding density of 5 × 10^4^ cells well^−1^. After trypsin digesting the attached cells after 4 h of seeding, cell numbers were counted using a hemocytometer. Cells on culture dish (polystyrene) were used as negative control. Cell adhesion rate was calculated from the proportion of adherent/seeded cells, and then normalized with control group. For cell proliferation test, cells were seeded on sterilized substrates in 24-well plates at 5 × 10^3^ cells/100 μl medium in each well. After incubating the cells in a humidified atmosphere with 5% CO_2_ at 37 °C for 3, 7, and 14 days respectively, cell proliferation reagent WST-1 (Roche, Switzerland) was added to the cells with 400 μL/well after medium change for 4 h incubation at 37 °C. The absorbance was then measured in 96 well plates by microplate reader (Bio-RAD 680) at 450 nm with a reference wavelength at 630 nm. Alkaline phosphatase (ALP) activity of osteoblasts was examined at 14 and 21 days using colorimetry-based assays. 400 μl mixed working solution was added to the lysed solution for 45 min, which consists of 100 μl 0.1 mM p-Nitrophenylphosphate, 1 ml 1 M MgCl_2_ and balanced carbonate buffer within 10 ml. Eventually, 100 μl 1 M NaOH was added to stop the reaction. ALP activity was evaluated as the amount of nitrophenol released through the enzymatic reaction and measured in 96 well plates at 405 nm using an ELISA reader.

### SILAC Labeling

All SILAC reagents were from the Pierce™ SILAC Protein Quantitation Kits (Thermo Fisher Scientific Inc, Waltham, MA). Cells were grown in SILAC “light” (L-lysine and L-arginine) and “heavy” (L-^13^C_6_-lysine and L-^13^C_6_^15^N_4_-arginine) conditions for at least five rounds of cell division in SILAC DMEM supplemented with 10% dialyzed fetal bovine serum for complete incorporation of isotope-labeled amino acids. In order to confirm that SILAC medium had not induced additional effect on the biocompatibility, cells in both “heavy” and “light” medium were examined with an optical microscope prior to the treatment. Subsequently, cells labeled with the “heavy” label were seeded onto n-HA/PEEK disks while cells with the “light” label were cultured on PEEK as control.

### Protein Extraction, Separation and In-gel Digestion

After 14 days of culture on both materials in SILAC medium, the cells were lysed and harvested in a buffer containing 7 M urea, 2 M thiourea, 4% w/v 3-[(3-Cholamidopropyl) dimethylammonio] propanesulfonate (CHAPS), 65 mMdithiothreitol (DTT), 1% (v/v) protease inhibitor cocktail (Sigma, USA), and 0.1 volume of the mixture of Deoxyribonuclease I (1 mg/mL) and Ribonuclease A (0.25 mg/mL) at 4 °C for 60 min. The lysates were dissolved with repeated vortex and ultra-sonication, followed by centrifugation at 15,000 g at 4 °C for 30 min to remove insoluble substances. The total protein concentration was determined by the Bradford assay. Proteins extracted from the surfaces of PEEK and n-HA/PEEK respectively were mixed at a ratio of 1:1 for a total of 60 μg proteins. They were then separated by sodium dodecyl sulfate polyacrylamide gel electrophoresis on a 12.5% gel. Gels were fixed and stained with Coomassie Brilliant Blue. Each gel lane was excised into 24 slices. The slices were then cut into 1 mm^3^ pieces and de-stained in 50% acetonitrile with 25 mM ammonium bicarbonate solution, before being dehydrated in 100% acetonitrile and dried. The in-gel proteins were reduced by incubation with 10 mM dithiothreitol for 40 min at 56 °C, followed by alkylation with 55 mM iodoacetamide for 40 min in the dark. After being washed and dehydrated, the proteins were digested with 8 ng/μL sequencing grade trypsin at 37 °C overnight. The peptides were extracted from gel pieces with 0.1% formic acid and 50% acetonitrile for 120 min twice, and the extracts were dried in a vacuum centrifuge.

### Liquid Chromatography-Tandem Mass Spectrometry (LC-MS/MS) Analysis and Data Acquisition

Peptides were re-dissolved in 10 μL 0.2% formic acid and injected into a fused silica emitter via auto-sampler with a commercial C18 reverse-phase column (150 mm long, 75 μm inner diameter, packed in-house with C18-AQ 5 μm resin), before being eluted with nano-flow liquid chromatography system (Micro-Tech Scientific, USA). This procedure used a linear gradient from 5% to 30% of acetonitrile in 0.1% formic acid at a constant flow rate of 500 nL/min over 110 min. Eluted peptides were sprayed into a linear ion trap (LTQ)-Orbitrap mass spectrometer (Thermo Fisher Scientific Inc., USA) via a nano-electrospray ion source.

The data was acquired in the positive ion mode and in a data-dependent mode to automatically switch between the full-scan MS (from m/z 300 to 2000) and the tandem mass spectra (MS/MS) acquisition. The survey spectra were acquired in the Orbitrap with the resolution set to a value of 60,000. Fragmentation in the LTQ was performed by collision-induced dissociation (CID) with 35% normalized collision energy.

Raw MS spectra were processed using the MaxQuant software (version1.0.12.31) that does peak list generation, quantitation, and data filtration and presentation^25^. MS/MS spectra were searched by Mascot (version 2.2.2, Matrix Science) against the IPI human data base (version 3.24) and the reversed sequences of all proteins. Enzyme specificity was set to trypsin. Carbamidomethylation was set as fixed modification, as well as L-^13^C_6_-lysine (Lys8) and L-^13^C_6_^15^N_4_-arginine (Arg10) (SILAC label modification). Variable modifications include oxidation (M) and N-acetylation (protein). Maximum two missed cleavages are allowed. Initial mass deviation of precursor ion and fragment ions was set up to 10 ppm and 0.5 Da. The minimum required peptide length was set to 6 amino acids. Protein identification requires two peptides, one of which has to be unique to the protein group. A false discovery rate (FDR) of 0.01 for proteins and peptides is required. False discovery rate of proteins is the product of the posterior error probability of the contained peptides where only peptides with distinct sequences are taken into account. Posterior error probability for peptides was calculated by recording Mascot score and peptide sequence length-dependent histograms of forward and reverse hits separately. Bayes theorem was then used to derive the probability of a false identification for a given top scoring peptide. False discovery rate was calculated by successively including best scoring peptide hits until the list contained 1% reverse hits. The procedure of protein expression quantification was described in detail in the reference^26^.

### Data Analysis and Interpretation

The protein ratios were normalized to correct unequal protein amounts to create normalized ratios of heavy/light proteins. In the experiment comparing n-HA/PEEK against PEEK, the quantified proteins were divided into three quantiles corresponding to the cutoffs of 2.0 and 0.5 of the normalized heavy/light ratios. Fold-changes >2.0 were considered as up-regulation, while <0.5 (reciprocal of 2.0)-fold changes were considered as down-regulation. Values between 0.5 and 2.0 indicated no change. Pearson’s correlation analysis was treated to different replicates. The enrichment and clustering analysis of different ratio quantiles for the Kyoto Encyclopedia of genes and genomes (KEGG) pathway and gene ontology (GO) biological process were performed separately by the Database for Annotation, Visualization and Integrated Discovery (DAVID, v6.7) system^27^,^28^ (http://david.abcc.ncifcrf.gov/). To understand the interaction of differentially expressed proteins visually, some biological pathways and networks were further analyzed by GenMAPP^29^ (http://www.genmapp.org/).

### Western Blot Analysis

Cells were lysed with the same method of protein extraction section above at day 14. An aliquot containing 30 μg of proteins were loaded onto 12.5% Sodium sodium-dodecyl sulphate polyacrylamide gels (SDS-PAGE), separated, and then transferred to polyvinylidene fluoride (PVDF) membranes. The membranes were then blocked in 5% skim milk and incubated with primary antibodies (Abcam, Cambridge, UK) against Filamins (FLNB), Clathrin (CLTC), Sarcoplasmic/endoplasmic reticulum calcium ATPase 2 (ATP2A2) and glyceraldehyde-3-phosphate dehydrogenase (GAPDH). The blots were developed by chem.-iluminescence using Amersham ECL reagents (GE Healthcare, Little Chalfont, UK).

### Intracellular Ca^2+^ Concentration Measurement

A Fluo-3/AM Calcium Assay kit (Beyotime, Haimen, China) was used to measure intracellular Ca^2+^ concentration following the manufacturer’s protocols. Briefly, after washing and trypsinization of the attached cells, the collected cells were incubated with 10 mM Fluo-3 dye solution containing probenecid to prevent extrusion of the dye out of cells at 37 °C for 30 min and then at room temperature for another 30 min. The fluorescent intensity of Fluo-3 was measured by flow cytometer (BD FACSCalibur, BD Biosciences, USA). The assay was done at 525 nm for excitation and at 530 nm for emission.

### Statistical Analysis

All experiments were done on six pieces of disc samples (n = 6). Data were averaged and expressed as mean ± standard deviation (SD). They were analyzed with ANOVA and the level of statistical significance was defined as p < 0.05.

## Results and Discussion

### Material Characterization

**Figure 1:**
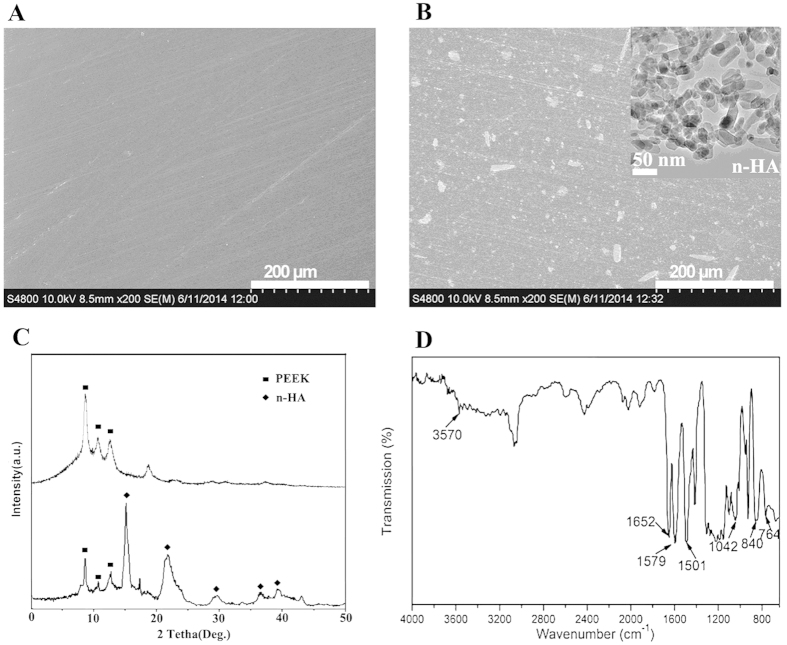
(**A**) SEM surface images of PEEK × 200; (**B**) n-HA/PEEK × 200; (**C**) XRD patterns of PEEK (the upper one) and n-HA/PEEK (the lower one) surfaces; (**D**) Fourier transform infrared scan of n-HA/PEEK.

SEM images of the polished surface topology are shown in Fig. 1A,B. Compare with pure PEEK (Fig. 1A), there are many white spots on the surface of n-HA/PEEK (Fig. 1B). These spots indicate the presence of HA on the surface of material. X-ray diffraction (XRD) patterns of the PEEK (the upper one) and n-HA/PEEK (the lower one) are shown in Fig. 1C. The peaks of 2θ at 18.71°, 20.76°, and 22.81° are characteristic peaks of PEEK. And the peaks at 25.9°, 31.8°, 40.0°, 46.7° and 49.5° are characteristic peaks of HA. No new peak appear in the n-HA/PEEK pattern except the inherent peaks of PEEK and HA themselves. This illustrates that there isn’t any new crystalline phase formation. Fourier transform infrared spectroscopy has confirmed the presence of an apatite phase in n-HA/PEEK, as shown in Fig. 1D. The strong peak at 1652 cm^−1^ originates from the C=O carbonylstretching vibration. The bands at 1597 cm^−1^ and 1501 cm^−1^ are in-plane vibration bands of benzene. Two bands of C–H vibration at 840 cm^−1^ and 764 cm^−1^ belong to the divided bands of the out-of-plane bending vibration absorption of benzene. Among them 840 cm^−1^ is from opposite-substituted aromatic ring. These are all characteristic peaks of PEEK. The bands of P-O stretch and vibration at 1042 cm^−1^ results from the PO4 group. The absorption peak at about 3570 cm^−1^ can be attributed to the stretching vibration of hydroxyl resulted from HA. The results of FT-IR combined with XRD confirm the composition of the composite material. The water contact angle of n-HA/PEEK is 73.43 ± 3.65 (^◦^) which is lower than that of PEEK 89.38 ± 4.07 (^◦^), suggesting higher hydrophilicity at the surface of n-HA/PEEK due to the presence of HA.

**Figure 2:**
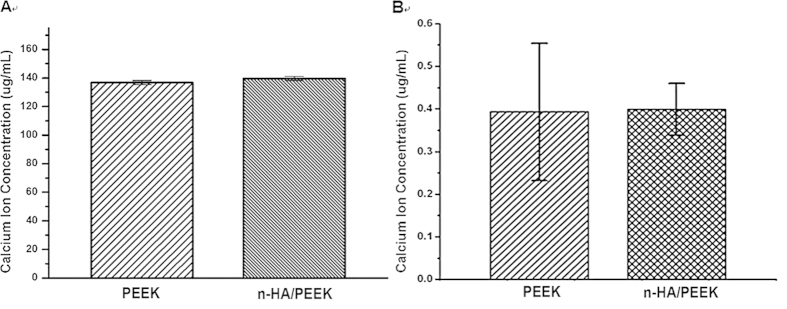
(**A**) Calcium concentrations in extraction media on PEEK and n-HA/PEEK substrates after incubation; (**B**) Calcium concentrations in PBS on PEEK and n-HA/PEEK substrates after incubation.

Direct addition of free HA nanoparticles has obvious proliferation inhibition effect even though HA is well known bio-active material^20^. An elevated concentration of calcium, resulting from dissolution of HA particles, has been shown to lead to decreased proliferation of mesenchymal stem cells^30^. Nanosized HA may lead to higher Ca^2+^ concentration of culture media compared with its conventional counterpart. So the toxicity mechanism may be attributed to calcium ion concentration. In n-HA/PEEK composite, the HA particles are embedded in PEEK rather than free. In order to investigate the calcium ion releasing behavior from n-HA/PEEK composite for a relatively long time, calcium concentrations of the culture solution extracts were examined. The results showed in Fig. 2A indicate no significant difference of Ca^2+^ concentration on surfaces of these two materials. Because there is high concentration of Ca^2+^ in the culture medium, the Ca^2+^ concentration in PBS condition was examined (Fig. 2B). The results suggest that the Ca^2+^ releasing amount of n-HA/PEEK is very low and no significant difference exists between these two materials. Thus free calcium ion in the solution environment only plays a minimal role in bio-effect of n-HA/PEEK.

### Cell Adhesion, Proliferation and Differentiation Assay

**Figure 3:**
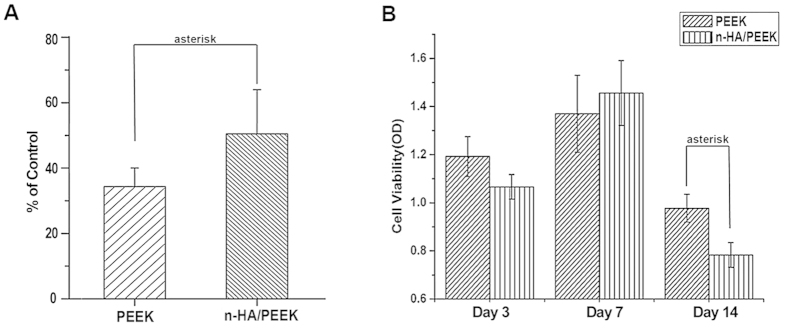
Cell attachment (**A**) and proliferation (**B**) of MG 63 cultured on PEEK and n-HA/PEEK. Columns marked with the asterisk, represent significant difference (p < 0.05) for the n-HA/PEEK group compared to the PEEK group.

**Figure 4:**
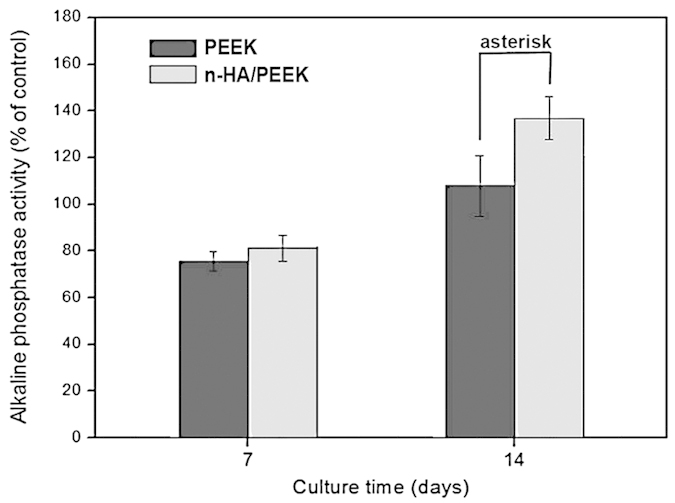
Alkaline phosphatase (ALP) activity of MG-63 cultured on PEEK and n-HA/PEEK. Columns marked with the asterisk, represent significant difference (p < 0.05) for the n-HA/PEEK group compared to the PEEK group.

Cytocompatibility assay was carried out to study cell attachment and proliferation using MG-63 cells. Cell attachment test results in Fig. 3A demonstrate that the number of cells attached to n-HA/PEEK surfaces is significantly higher than that to PEEK. It may be related to the higher hydrophilicity of n-HA/PEEK. Cell proliferation results in Fig. 3B show that cells cultured on n-HA/PEEK surface have a significant lower proliferation ratio than PEEK surface after 14 days of culture. Alkaline phosphatase (ALP) activity is a characteristic index of osteoblast differentiation^31^. The results in Fig. 4 show significant difference between PEEK and n-HA/PEEK materials at day 14 (the control indicated cells on culture plates). This suggests that HA component on the surface of material have improved alkaline phosphatase (ALP) activity of PEEK. It is reported that skeletal development requires the correct balance of osteoblast proliferation, survival, and differentiation which is modulated by a network of signaling pathways and transcription factors^32^. Maybe at day 14, the lower level of proliferation is correlated with the higher degree of differentiation.

### Quantitative Proteomic Comparison Results

**Figure 5:**
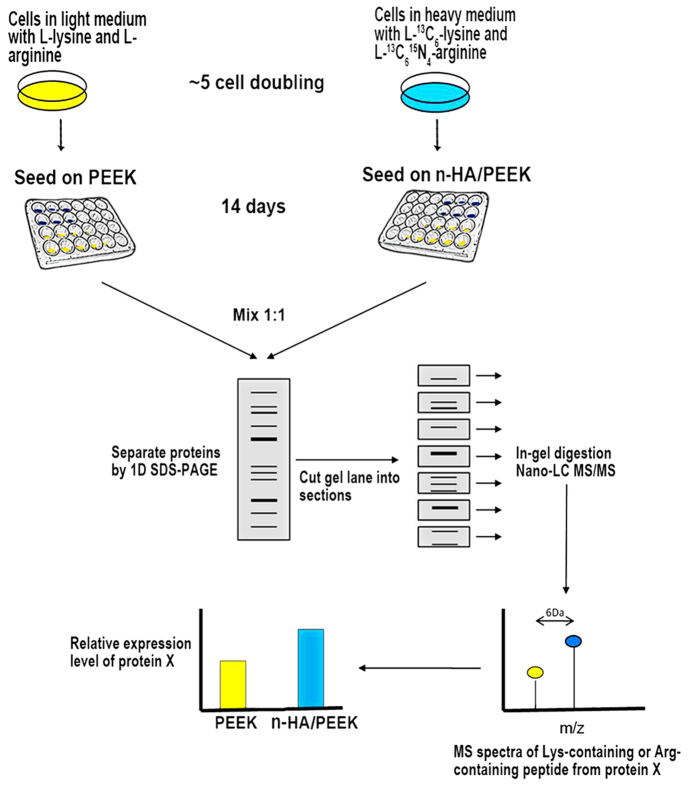
Flowchart of SILAC coupled with LC-MS/MS for the comparative analysis of protein expression in the MG-63 cells upon PEEK and n-HA-PEEK treatment.

**Figure 6:**
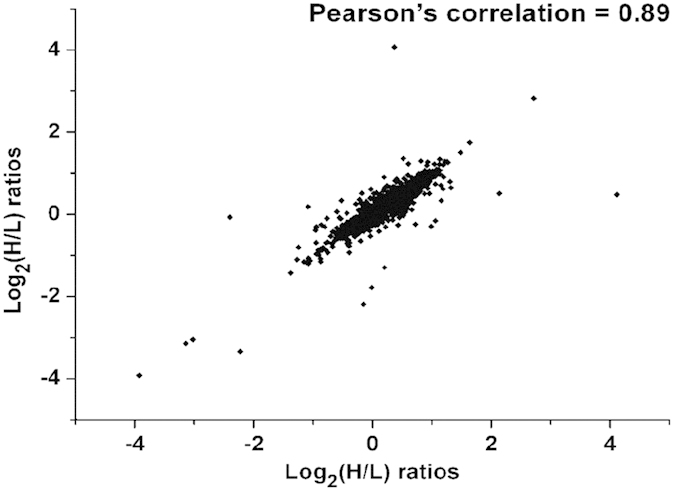
Quantitative reproducibility analysis of duplicated experiments by Pearson’s correlation.

The obvious difference of cell proliferation between these two materials on day 14 suggests that day 14 is an important time point for biocompatibility. ALP activity results also show that a significant functional difference appears on day 14. To investigate the molecular mechanism behind the difference of these phenomena, quantitative proteomics study was performed after 14 days of cell culture on these two surfaces. In order to characterize the alterations in global protein expression of osteoblast proteome in response to direct contact with n-HA/PEEK compared with PEEK, MG-63 cells were labeled and the proteins were quantified following standard SILAC operating procedures (Fig. 5). Two biological replicate experiments were carried out, and a Pearson’s correlation analysis was used to assess the correlation between these two datasets. Excellent quantitative reproducibility was obtained (Pearson’s correlation coefficient 0.89) (Fig. 6). The two datasets were then combined and analyzed together using stringent and unified criteria which is available in MaxQuant^33^.

**Figure 7:**
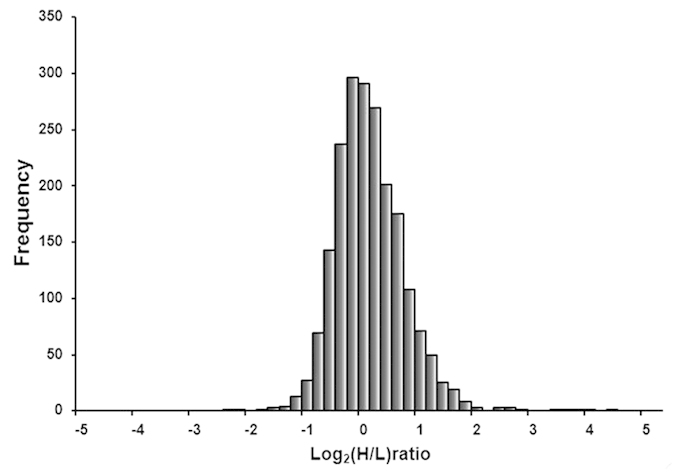
Fold-change distribution of the proteome (H: n-HA/PEEK, L: PEEK). Quantitative comparison of MG-63 proteome on n-HA/PEEK and PEEK surfaces.

Histograms shown in Fig. 7 indicate that there is little overall change in the cells proteome, and the number of up-regulated protein is a little more than down-regulated protein. At a false positive rate of less than one percent, a total of 2303 proteins were identified, and 2032 proteins were quantified. Detailed quantitative results are shown in Table S1. The fold difference that is greater than 2.0 or less than 0.5 is considered as significant. As a result, 118 proteins are up-regulated and 53 proteins are down-regulated in the cells treated with n-HA/PEEK compared to PEEK.

Functional classification and enrichment analysis were performed via the DAVID platform. The normalized ratios in both up- and down-regulation sections of the comparison were further classified separately and a statistical test was performed to assess the over-represented categories with reference to the databases of KEGG pathways and GO biological processes. The categories with a *p* value less than 0.05 were maintained.

Table 1 shows that many enriched categories in the up-regulated proteins are related to processes involving calcium ion, such as “Endocrine and other factor-regulated calcium reabsorption”, “Calcium signaling pathway”, “Calcium ion transmembrane transport” and “Sarcoplasmic reticulum calcium ion transport”. Furthermore, some categories associated with functions of calcium, such as “Mineral absorption”, “Ion transport” and “Regulation of actin cytoskeleton”^34^ were also enriched. HA in this study is imbedded in composite rather than free. Even though there is no covalent bond formation between HA and the matrix PEEK, the release of calcium ion into the medium incubated with HA–PEEK is not significantly increased. However, from the proteomic analysis results, the calcium on the surface of material still has influence on the intracellular calcium ion related pathway and function. Calcium ion participates in numerous important physiological processes including intracellular signaling processes, neuronal excitability, muscle contraction and bone formation. Some of the processes need to be promoted, like mineralization in the bone repair. Nevertheless, the disturbing of normal intracellular calcium ion level may lead to disorder in critical physiological function.

**Figure 8:**
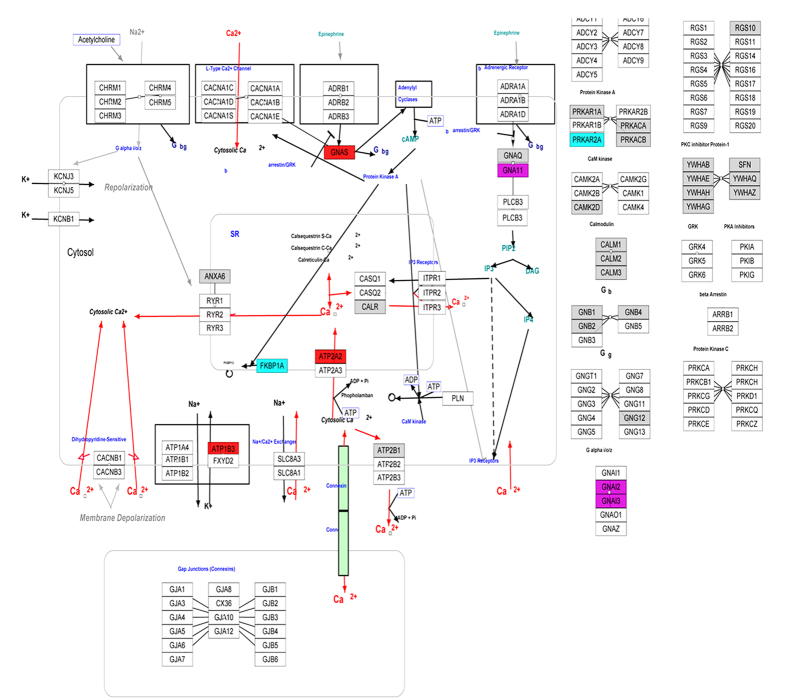
The calcium regulation pathway ( http://www.genmapp.org) in Homo sapiens. Each box represents a protein, within which is the symbol of the gene which encodes the protein. Arrows show the process of the pathway. Red: up-regulated more than 2.0-fold; Fuchsia: up-regulated between 1.5- and 2.0-fold; light blue: down-regulated between 1.5- and 2.0-fold; dark blue: down-regulated more than 2.0-fold; gray: no sense in expression change; blank: not detected in this study.

**Figure 9:**
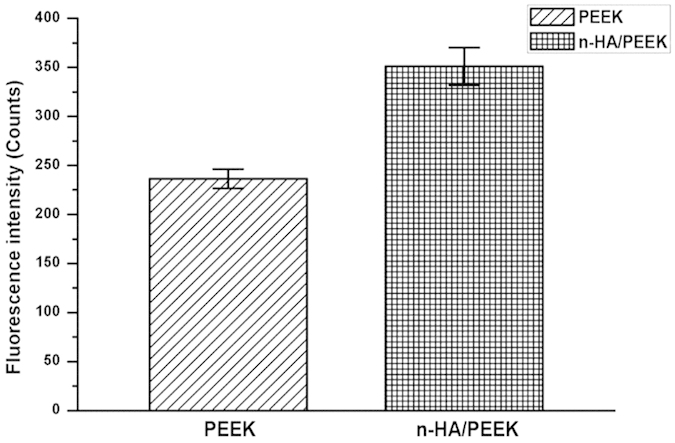
The intracellular calcium levels in MG-63 cells cultured on surfaces of PEEK and n-HA/PEEK. The difference is significant (p < 0.05) for the n-HA/PEEK group compared to the PEEK group.

To further understand the crucial process, which are most enriched and many differentially expressed proteins closely relate to, the biological pathway “the calcium regulation pathway” is illustrated in Fig. 8 by GenMAPP software. It was found that besides the proteins with no change (gray boxes), most differentially expressed proteins which were captured in the proteomic detection were up-regulated. And only a small number of slightly down-regulated proteins (between 1.5- and 2.0-fold) were detected. The enrichment results suggest that the signals from HA could modulate a series of proteins involved with calcium. The addition of HA may not greatly affect the culture media ion concentrations but could have influence to microenvironment around the substrate, which in turn may influence the intracellular calcium ions in the cells on the disk. Quantification for the ratios of intracellular calcium concentrations in the osteoblast cells after culturing for 14 days in respond to n-HA/PEEK is showed in Fig. 9, which further supports the explanation.

In addition, “Focal adhesion” showed enrichment in the up-regulated proteins. Focal adhesion is specialized structures formed at the cell–extracellular matrix (ECM) contact points that connect the cell cytoskeleton to the ECM, and they play roles in cell adhesion, cell proliferation, and cell differentiation^35^,^36^. From the result of cell attachment and differentiation test, n-HA/PEEK composite enhanced the cell adhesion and differentiation of osteoblast. These cell behaviors also have relation to calcium signaling^37^,^38^. And from the quantitative proteomic results, the molecular mechanism about the differences of these aspects can be traced.

In the down-regulated proteins, it was found that except the “Ribosome”, the most enriched categories are about processes related to RNA process (Table 2), like “Spliceosome”, “mRNA surveillance pathway”, “RNA transport”, “RNA splicing” and “mRNA 3′-end processing”. The left categories like gene expression and translational elongation also have relationship with RNA. Enrichment in the down-regulated proteins suggests that n-HA/PEEK composite has negative effect to cell proliferation, and the down-regulation of these processes may also be one of the reasons for cell proliferation result on day 14 described above.

**Figure 10:**
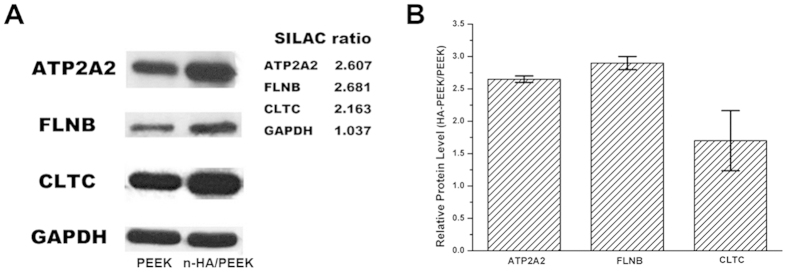
Western blotting validation of the SILAC quantitative results about notable proteins. (**A**) Blots representatives of at least three independent experiments. The right side shows the SILAC ratios of each protein. (**B**) Densitometry analysis of the western blots. The intensities of each test protein bands were normalized against the corresponding GAPDH blot bands.

Several proteins we concentrated on were confirmed by western blotting analysis (Fig. 10). Western blots were quantified using Image J software. The bar graph shows the intensities of results, and the intensities of each test protein band were normalized against corresponding GAPDH blot bands. From the result of functional enrichment analysis it can be seen that some differentially expressed proteins are associated with calcium function. Sarcoplasmic/endoplasmic reticulum calcium ATPase 2 (SERCA2, gene name ATP2A2), also named calcium pump 2, is a magnesium-dependent enzyme. It catalyzes the hydrolysis of ATP coupled with the translocation of calcium from the cytosol to the sarcoplasmic reticulum lumen. Isoform 2 is involved in the regulation of the contraction/relaxation cycle^39^. It is a sarco (endo) plasmic reticulum Ca^2+^-ATPase which pumps Ca^2+^ into the inositol-1,4,5-triphosphate-sensitive stores, and it is involved in the maintenance of intracellular Ca^2+^ homeostasis as well as in G-protein-coupled signaling events^40^. It catalyzes this reaction: ATP + H_2_O + Ca^2+^ (Side 1) = ADP + phosphate + Ca^2+^ (Side 2). This protein is involved in the following biological processes: Calcium transport, Ion transport, cellular calcium ion homeostasis^41^,^42^, cell adhesion^43^, blood coagulation, epidermis development, sarcoplasmic reticulum calcium ion transport^41^,^44^ and so on. It also has molecular function like ATP binding and calcium-transporting ATPase activity^41^. Lack of it is the cause of Darier disease^45^. It was found that a disruption of SERCA2 expression has critical roles in osteoclast differentiation and impairs osteoclastogenesis^46^. However, major expression of isoform b of SERCA2 was demonstrated in normal osteoblasts^47^. In this study, this protein was obviously up regulated. Except the function about calcium ion, the mechanism of composited n-HA’s effect on osteoblast differentiation might be via SERCA2-dependent Ca^2+^ signaling. Filamin B (FLNB) connects cell membrane constituents to the actin cytoskeleton. It may promote orthogonal branching of actin filaments and link actin filaments to membrane glycoproteins^48^,^49^. It also anchors various transmembrane proteins to the actin cytoskeleton^50^.

FLNs are actin-binding proteins that organize actin filaments into parallel arrays or three-dimensional webs, linking them to cellular membrane^51^. This protein is involved in the following biological processes: cell differentiation, actin cytoskeleton organization^52^, cytokine-mediated signaling pathway, cytoskeletal anchoring at plasma membrane^49^, and molecular function of actin binding^52^. Clathrin (CLTC) is the major protein of the polyhedral coat of coated pits and vesicles^53^. CLTC is involved in the following biological processes: cellular membrane organization, intracellular protein transport^54^, post-Golgi vesicle-mediated transport, receptor internalization and transferrin transport^55^. In addition, collagen VI which correlated with cell-binding and cell adhesion was also included in the differential proteins. These proteins all showed up-regulation in this study compared with pure PEEK, which suggested or verified that the existence of n-HA on the surface of composite material have promoted the cell adhesion, differentiation and calcium ion transport.

**Figure 11:**
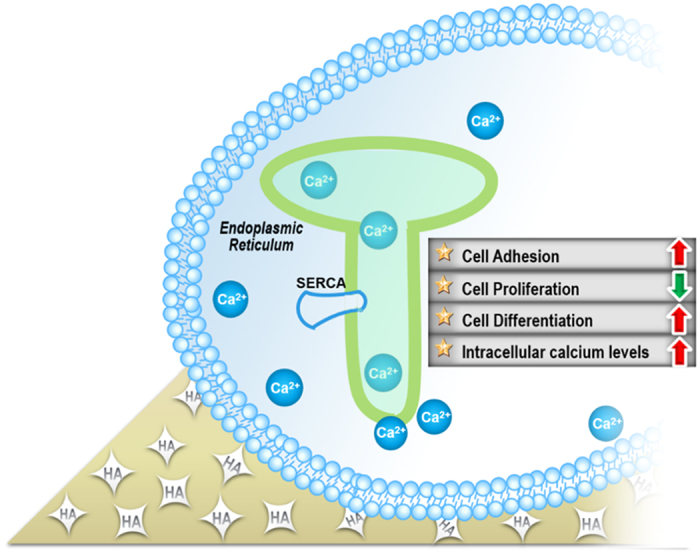
Scheme of biological effect and molecular mechanism by n-HA/PEEK composite.

By quantitative proteomic analysis, we discovered some subtle response of osteoblast and explained how the cellular level results were obtained from the molecular aspects. The scheme of the major biological effects induced by n-HA/PEEK composite is shown in Fig. 11. PEEK is generally considered as bio-inert material^3^, and the additive HA is a well-known bio-active constituent^9^. Although HA was mixed in the PEEK in the study, the calcium ion level hadn’t increased in the culture medium. And the influence to intracellular calcium related processes may come from the direct contact between cells and calcium fixed on the surface of material. Except the promotion of osteoblast differentiation potential, the proliferation inhibition effect that was found in free n-HA^20^ particles still exist in the composition state. n-HA/PEEK was expected to be nontoxic, friendly and pure bio-active, But the study result is different from our existing consideration to this composite material. In addition, the implant produce debris after implantation to humans or animals is possible. There hasn’t *in vivo* studies of n-HA/PEEK reporting this aspect yet. But a review about the harm of implant debris has reported that the smaller-sized debris are more pro-inflammatory by virtue of their greater numbers (dose) for a given amount of implant mass loss^56^. Although this reporter also said that metal particles are more pro-inflammatory than polymers, n-HA/PEEK may has to draw more attention in the debris issue.

### Study Limitations

The object of this current study is need to be expanded and there still have many aspects to explore in the further study. For example, the highest proportion of n-HA was used in this study, but whether the cell inhibition effect has dose-effect relationship is not well known, because the calcium ion dissolution hasn’t been detected in such high concentration of n-HA in the composite. And whether the direct contact effect is related with the amount of HA is need to study. Moreover, if the rough surface (not polish) was used, then some calcium ion might be dissolved to the medium. In that case, the biological effect may be different. At diverse roughness, the results will be more complicated, and that would be much more work for proteomic analysis. Even so, certain roughness is need in the application of bone implant material. The research is always need to be approaching the final application state from the standard and simple beginning. Anyway, the proteomic study creates a new scope to in-depth understanding to the biological effect of implant materials.

## Conclusions

Through the quantitative proteomic analysis, the global protein expression of osteoblast behind the comprehensive biological effects of n-HA/PEEK has been studied in comparison with pure PEEK. It was found that the number of cells attached to n-HA/PEEK surfaces is significantly higher than that to PEEK. Cells cultured on n-HA/PEEK surface have a significant lower proliferation ratio than PEEK surface after 14 days of culture. Alkaline phosphatase (ALP) activity evaluation suggests that HA component on the surface of material have improved ALP activity of PEEK. Results from quantitative proteomic analysis show that the most enriched categories in the up-regulated proteins are related to calcium ion processes and associated functions while the most enriched categories in the down-regulated proteins are related to RNA process. The intracellular Ca^2+^ concentration are higher for n-HA/PEEK than pure PEEK. There is no increase of calcium ion level in the incubated medium. Therefore, the influence on intracellular calcium related processes is mainly attributed to the calcium existed on the surface of n-HA/PEEK, which may contribute to the intracellular Ca^2+^. These findings may explain the molecular mechanism of the biological functions of the acceleration of adhesion, differentiation and the inhibition of proliferation of n-HA/PEEK.

## Additional Information

**How to cite this article**: Zhao, M. *et al*. Response of Human Osteoblast to n-HA/PEEK—Quantitative Proteomic Study of Bio-effects of Nano-Hydroxyapatite Composite. *Sci. Rep.* **6**, 22832; doi: 10.1038/srep22832 (2016).

## Supplementary Material

Supplementary Information

## Figures and Tables

**Table 1:** Functional annotation categories in the enrichment results of over-expressed proteins.

Category	Term	Count	*p*-Value
GO_BP	GO:0055085 Transmembrane transport	23	4.19081E-15
GO_BP	GO:0006886 Intracellular protein transport	13	2.45E-11
GO_BP	GO:0006810 Transport	18	2.17E-10
KEGG	04961: Endocrine and other factor-regulated calcium reabsorption	7	7.41639e-09
KEGG	04978: Mineral absorption	3	3.73045e-05
KEGG	04974: Protein digestion and absorption	6	1.93344e-05
GO_BP	GO:0006811 Ion transport	10	1.37E-05
KEGG	04020: Calcium signaling pathway	3	0.000241344
KEGG	04510: Focal adhesion	6	0.000503433
GO_BP	GO:0070588 Calcium ion transmembrane transport	2	0.00589087
GO_BP	GO:0070296 Sarcoplasmic reticulum	1	0.00653755
Calcium ion transport			
KEGG	Regulation of actin cytoskeleton	6	0.00806353

**Table 2:** Notable functional annotation categories in the enrichment results of under-expressed proteins.

Category	Term	Count	*p*-Value
GO_BP	GO: 0010467 gene expression	11	1.08E-09
GO_BP	GO: 0000398 nuclear mRNA splicing, via spliceosome	8	6.37E-09
GO_BP	GO:0008380 RNA splicing	8	1.76E-07
KEGG	03010: Ribosome	5	4.03E-07
KEGG	03040: Spliceosome	5	9.81E-07
GO_BP	GO:0006415 translational termination	5	3.86E-06
GO_BP	GO:0031124 mRNA 3′-end processing	4	4.38E-06
GO_BP	GO:0006414 translational elongation	5	4.72E-06
KEGG	03015: mRNA surveillance pathway	4	4.87E-06
KEGG	03013: RNA transport	3	0.000929054
